# The effectiveness of music–movement integration for vulnerable groups: a systematic literature review

**DOI:** 10.3389/fpsyg.2023.1127654

**Published:** 2023-08-31

**Authors:** Marja-Leena Juntunen, Katja Sutela

**Affiliations:** ^1^Department of Music Education, Sibelius Academy, University of the Arts Helsinki, Helsinki, Finland; ^2^Faculty of Education, University of Oulu, Oulu, Finland

**Keywords:** music-movement integration, Dalcroze, vulnerable, special needs, older adults, systematic literature review

## Abstract

This systematic review synthesized the outcomes of previous intervention studies published from January 2000–October 2022 to evaluate the effectiveness of Dalcroze-based or similar music–movement integration among groups of individuals considered vulnerable (in relation to their abilities and health/wellbeing). The target groups addressed in previous intervention studies included individuals with special educational needs (such as disorders, disabilities, or impairments) or with a (risk of) decline in health and/or physical strength. Twenty articles met the review inclusion criteria. All studies showed beneficial outcomes for music–movement intervention except one that suffered from low adherence rates. In older adults, the benefits were cognitive, physical, social, and/or emotional, including improved postural stability, balance, gait safety, confidence in mobility, metamemory skills, dual-task performance, social and physical pleasure, autotelic/flow experience, enjoyment, health, and quality of life. In individuals with special educational needs, improvement was seen in relation to inclusion, reductions in compulsive and other problematic behaviors, self-regulation, perceptual and cognitive abilities and functions, linguistic and learning skills, auditory attention and phonological awareness, social interaction, engagement, and agency.

## Introduction

This systematic review examined and synthesized intervention studies published from 2000–October 2022 that investigated the effectiveness of Dalcroze-based (or similar) music–movement integration in a variety of contexts among “vulnerable groups.” “Dalcroze-based” here refers to practices of music–movement integration in which whole-body movement is a way of responding to, expressing, and experiencing music rather than an end in itself (as opposed to in dance where the role of music varies greatly). Vulnerability as a notion is complex and, as Schröder-Butterfill and Marianti (2006, p. 9) noted, contains substantial conceptual and terminological diversity. Etymologically, vulnerability refers “to the susceptibility of being physically or emotionally wounded” (Sanchini et al., 2022). Vulnerability can be defined both as resulting from a condition and a process related, for example, to wellbeing, the possibility of exposure to risk, and capacity to manage risks (Zarowsky et al., 2013). It has been suggested that vulnerability does not depend on any single attribution, but rather it results from complex relationships among a variety of factors, such as social class, gender, race, age, and ability—people are made vulnerable rather than born vulnerable (Kuran et al., 2020). Thus, being vulnerable is not necessarily a fixed existential position (Zarowsky et al., 2013), and there are different bases for defining who can be considered vulnerable. In this article, our working definition of vulnerable groups draws on a more traditional and narrow view related to ability- and health/wellbeing-related factors.

Thus, by vulnerable we here refer to groups of individuals whose possibilities for participation, learning, and/or agency in music and beyond are (at risk of being) restricted (see Ketefian, 2015, p. 165) as a result of having (or being at risk of having) a disorder, disability, impairment, or decline in health and/or physical strength (see Schröder-Butterfill and Marianti, 2006). Based on our preliminary examination of research articles according to these criteria, older adults, who have historically been associated with vulnerability (Sanchini et al., 2022) and people with special educational needs (SEN) were identified as the main target groups of Dalcroze-based music–movement interventions in the articles in the chosen time period. SEN implies learning difficulties or disabilities that require support in an additional or different form than what is generally available to others of a similar age (Florian, 2019, p. 693) and includes impairments (cognitive, memory, visual, and/or hearing), dyslexia, autism spectrum disorder (ASD), as well as developmental, behavioral, and/or cognitive challenges.

### Music–movement integration

In this study, the integration of music and movement as a practical activity not only refers to their co-existence but also to deeper reciprocal connections between music and movement, based on the shared characteristics of music and body/movement (see e.g., Sievers et al., 2013). The interconnection and shared qualities have been identified in several fields of science, such as ethnomusicology (Blacking, 1977), neuropsychology (e.g., Seitz, 2005a,b), the neurosciences (e.g., Hodges and Gruhn, 2012), and musicology (e.g., Lidov, 2005; Godøy, 2018). Many philosophical approaches within music education, such as the phenomenological approach (e.g., Bowman, 1998; Juntunen, 2004) and praxialism (e.g., Elliott, 2005), address the role of the body and movement in musical experience and knowledge.

Within the cognitive sciences, the enactivist approach in particular focuses on how concrete sensorimotor patterns of action and perception underlie and shape our cognitive processes and emphasize the role of the human body as a mediator for meaning formation (Leman and Maes, 2014; Gallagher, 2017, p. 5). Perception, action, and cognition are regarded as mutually dependent and deeply intermixed in our worldly experience (Schiavio, 2014). During the last decade, embodied music cognition research has become an influential paradigm treating embodied interactions with music in diverse ways (Leman et al., 2018). Among other things, it highlights how body movement and bodily interactions with music influence music perception and strongly determine music cognition (Leman and Maes, 2014; Leman et al., 2018). It has also been suggested that besides being connected on a behavioral level, sound and body motion are intertwined on a neurophysiological level (Kohler et al., 2002).

Today, movement is among the key working methods of many music education and therapy practices, and integrating (rhythmic) movement and music in educational and therapeutic practices has been identified as beneficial in many studies. The idea of music–movement integration in music education was first presented by Swiss musician, composer, and music educator Jaques-Dalcroze (1865–1950) in the late 19th century. His pedagogical efforts were aimed at how musical experience, understanding and learning could be more rooted in perception and embodied experience (Juntunen, 2022). Although he began with a focus on exploring and testing the possibilities of music–movement integration in the context of music education, he soon recognized its therapeutic potential, as the practice aims to reinforce the unity of the body, mind, and emotions (Jaques-Dalcroze, 1921/1980, p. 182; Habron, 2014). Consequently, by 1930, Dalcrozian ideas had been applied in many fields related to therapy (Dutoit, 1965, p. 67) and Dalcroze teachers employed ideas from the approach in their work with individuals with special needs. Such practices have continued to develop. At present, Dalcroze applications can be found in many practices, disciplines, and fields of research, such as music, dance, theater, cinema, special education, music therapy, and gerontology (Mathieu, 2010; Habron, 2014).

In Dalcroze-based teaching, participants in group activities respond through whole-body movement to (often improvised) music. Music is explored, experienced, learnt, and expressed through movements that show what the participants hear and understand of the music (Juntunen, 2016). The exercises can be functional (e.g., reflecting the dynamics of music), rhythmic, creative, and dramatic, among other possibilities (for more, see e.g., Juntunen, 2002, 2016, 2022). The approach addresses a wide range of capabilities on the physical, cognitive, affective, and social domains (Juntunen, 2016; Treviño and Álvarez-Bermúdez, 2018b). In Dalcroze practice, music–movement integration implies multisensory integration, which is also one of its recognized benefits (e.g., Altenmüller and Scholz, 2016; Juntunen, 2020a,b). Multisensory pertains to the integration of information from different sensory modalities. In music–movement integration, you actively listen and respond to the music, which guides your movement (showing what you hear), while simultaneously seeing other participants move and perceiving your movement through kinesthesia, both of which influence movement and listening.^1^ Another important and beneficial feature of Dalcroze training is that the movement is mainly improvisational and involves responding to music—while interacting with the movement of others—and thus it activates the so-called perception–action loop (for more, see e.g., Capdepuy et al., 2012; Schiavio, 2014; Spivey and Huette, 2014). It is precisely because of these qualities of music–movement integration that this study focused on Dalcroze-based practices (instead of dance, for example). Some of the ideas of the Dalcroze approach have been adopted and modified in other (music education) approaches, such as those of Laban, Orff, and Kodály (see Juntunen, 2022; also Abril, 2011). These approaches share similarities and differences in relation to the role of movement; however, each builds on the close connection between music and movement.

## Methods

### Aims and objectives

This systematic literature review summarizes and synthesizes the evidence for the effectiveness of Dalcroze-based (or similar) music–movement integration interventions among vulnerable persons and integrates evidence from quantitative, qualitative, and/or mixed-methods studies (see Mays et al., 2005; Pluye et al., 2009; Booth et al., 2016; Hong and Pluye, 2018). It also provides insights into the intervention practices and types of exercises used in the interventions.

To be included in the review, the target group had to be identified as vulnerable, according to our working definition, and the studies had to explicitly mention the use of both music and whole-body movement. For example, studies of music education and music therapy that did not involve the use of movement or in which body movement involved only stomping, tapping, playing an instrument, or similar were excluded. The studies that did not explicitly talk about music were also omitted.^2^ Furthermore, music had to be more than just (mechanical) rhythm. Thus, interventions that used only a metronome or another steady rhythmic pulse as music were not included. The studies had to be based on an intervention and apply valid measures. Finally, only journal articles reporting original research were included. Other forms of publications, such as book chapters, dissertations, textbooks, (systematic) reviews, letters, and conference proceedings and abstracts, as well as works based on the reflections of practitioners, were excluded from the review.

In short, the inclusion criteria for the journal articles were as follows: they had to (1) have been conducted with vulnerable individuals (according to our criteria), (2) be based on an intervention, (3) examine the influences of music–movement integration (explicitly mentioning music), (4) use reliable and valid measures, and (5) be published in a scientific journal, (6) be in English, and (7) have been published from January 2000–October 2022.

### Electronic searches

We selected the articles through a process of identification and conducted literature searches in the following eight databases: Academic Search Ultimate, Arsca, Finna, Google Scholar, ERIC, PsycInfo, PubMed, and Web of Science. The search terms below were employed in the databases:

(“deficiency” OR “disabled” OR “disorder” OR “special needs” OR “learning difficult*” OR “social anxiety” OR “seniors” OR “older adults”)

AND (“music education” OR “music therapy” OR “music intervention” OR “music movement therapy”)

AND (“Dalcroze” OR “music(–)movement” OR “music and movement”).

Additionally, the snowball technique and citation tracking were used to identify publications that met the criteria.

**FIGURE 1:**
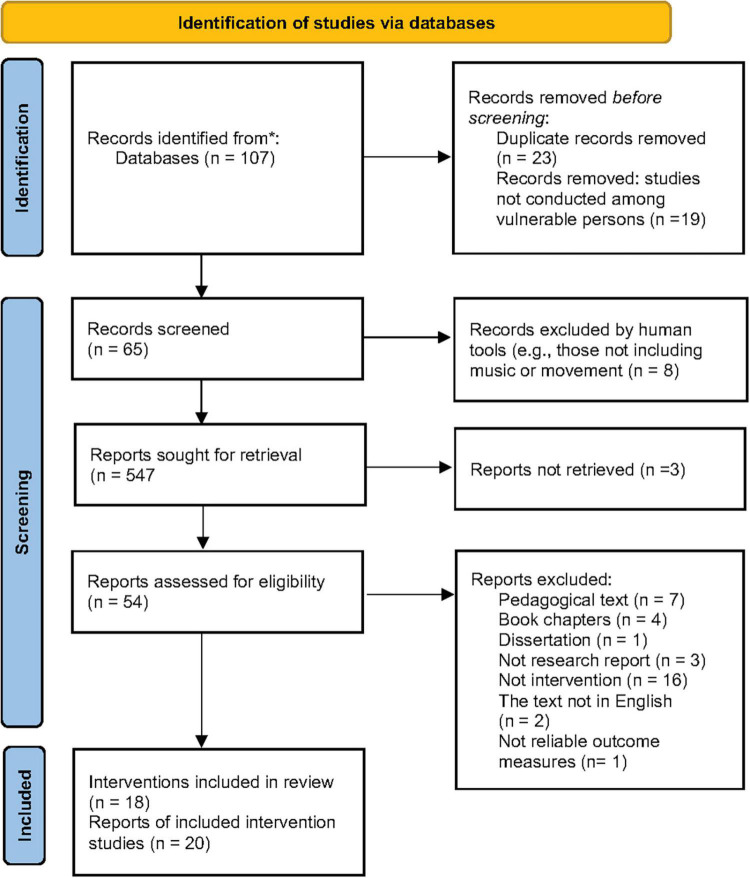
PRISMA flow diagram of the systematic literature review search illustrating the number of studies retrieved from the systematic search and the final number of studies included in the systematic review.

Before screening the articles, we removed duplicates and studies that were not conducted with vulnerable persons. Then, we excluded the articles that did not fulfill the inclusion criteria. Figure 1^3^ illustrates the number of studies retrieved from the systematic search and the final number of studies included in the review in the form of a PRISMA flow diagram.

Before the analysis, we generated a database by using Excel to collect, categorize, and save information about the articles, including author(s) and year of publication; title of the article; publication information; research task/question; research design; participants; context; type of music–movement exercises; process; measures; outcomes; and conclusion. We divided all the extracted articles that met the criteria into three groups: (1) individuals with special (educational) needs or impairment (*N* = 4); (2) individuals with dyslexia (*N* = 3); and (3) older adults with a risk of or suffering from a decline in health and/or physical strength (*N* = 10 + 3). Later, we combined the first two categories because dyslexia is considered a special need and classified as a learning disability.

### The review process

In the review, we used the SPIDER strategy (see Table 1), and also considering the topic of the journal number to which the review was to be submitted (*Music Education, Embodiment and Flourishing*), we formulated the following research question:

**TABLE 1:** The SPIDER strategy (as used in the review).

S	Sample	Vulnerable groups of individuals (as defined in the text)
P	Phenomena of interest	Dalcroze-based or similar music-movement integration interventions
D	Design	Published intervention studies in research journals
E	Evaluation of outcomes	Measured or otherwise identified beneficial influences of music-movement integration
R	Research type	Quantitative, qualitative, and/or mix- methods

*1. What were the outcomes of music–movement integration interventions among vulnerable groups?*

As we were also interested in how the outcomes were measured, what was done in the interventions, and what was found to be particularly beneficial in the interventions, we incorporated the following questions:

*2. What measures were used to assess the outcomes?*
*3. What types of exercises were included?*
*4. What qualities of music–movement integration were identified as effective?*

To answer these questions, we both summarized and synthesized the studies.

### Overview of the included studies

The data for the analysis consisted of 20 articles (based on 18 intervention studies). Twelve interventions were conducted among older adults (13 publications), including three studies among individuals with diagnosed cognitive decline or impairment (see Table 2). The interventions focused on the improvement of body posture, balance and gait, cognitive functions, autotelic and positive experiences, as well as wellbeing and quality of life more generally. Six interventions were conducted among individuals with varying SEN (seven publications): three among those with dyslexia, one among a mixed group of students with SEN, one among individuals with ASD, and one with brain injury patients (see Table 3). The interventions among people with SEN focused on supporting participants’ bodily and cognitive functions, linguistic skills, agency, interactions, and/or inclusion. In the reference list, the reviewed articles are marked with an asterisk.

**TABLE 2:** Studies of older adults.

References	Origin	Purpose	Research design/Methods	Target population	Context
Adamczyk et al., 2020	Poland	Investigate the impact of performing exercises involving both physical and mental spheres on the dynamic agility in older women	Intervention-control groups (IG: 34) and control (CG: 39); twice a week (45 min.), 12 weeks	Older women (69:9 ± 3:2); living in a community (*N* = 59)	Recruited via newspaper and internet: women aged 65+, community-dwelling, no contrains to participate in physical activities
Adamczyk et al., 2022	Poland	Investigate the effect of Dalcroze approach on the postural stability of women over 65 years of age	Randomized controlled trial—parallel group design, IG (*n* = 26) and twice a week (45 min.) f12 weeks; CG, *n* = 33)	See above (based on the same intervention)	See above
Beaulieu et al., 2017	USA	Examine older adults’ perceptions on the strengths and challenges of the Dalcroze programme, and their recommendations for making improvements in the next action research phase	Action research, 1,5 years, focus group interviews	Older adults, aged 65–94. 24 older adults participated in the programme, but about 25% dropped out in the first month, (*N* = 18), eight participants attended regularly through the first year and a half	A community health programme based on Dalcroze approach, in the San Marcos Senior Activity Center in California
Choo et al., 2020	New Zealand	Explore the effects of the intuitive movement reembodiment (IMR) program on the quality of life (QoL) of older adults with dementia	Pilot intervention for 3 groups: 1: mild dementia, 2: moderate dementia, 3: advanced dementia.10 weekly sessions, no control group	Older women, recruited (1) age 60–95, (2) physically able to take part in singing and dancing activities, (3) diagnosis of dementia by a trained health professional, (4) English as their first language, (*N* = 22)	Hospital New Zealand
Ferguson-Stegall et al., 2017	USA	Determine if a 3-mo Dalcroze intervention improves measures of gait and balance, self-perceptions of health, and fear of falling in a community-dwelling elderly cohort	Intervention, 6 months, no control group	Older adults, recruited 8 females, 1 male; age 79.0 ± 12.3 years) (*N* = 9)	Community senior program
Fischbacher et al., 2020	Switzerland	Evaluate the easibility, safety, and exploratory effectiveness of a Dalcroze program and a home exercise program designed for fall prevention in older adults with mild cognitive impairment (MCI) or early dementia. to test the feasibility of recruitment and safety of the interventions	Three-arm, single-blind, 12-month randomized controlled pilot trial: control group	Older adults (*N* = 60); yet only 18 participants could be enrolled; recruited community-dwelling women and men age 65 years and older with MCI or early dementia through participating memory clinics	Memory clinics in Zurich, Switzerland, recruitment through mailing lists and referrals from physicians of three memory clinics
Hars et al., 2014	Switzerland	Study the effects of long-term Dalcroze-based multitask exercises on physical function and fall risk in older adults	A 3-year follow-up extension of a 1-year randomized controlled trial (Trombetti et al., 2011), four years following original trial enrolment, control group	Community-dwellers aged ≥ 65 years at increased risk of falls (*N* = 134)	Music-based multitask exercise program Geneva, recruited participants: (1) aged 65 + , (2) living in the community, (3) without Dalcroze experience (except in childhood), (4) at increased risk for falling
Kressig et al., 2005	Switzerland	Compare stride-to-stride variations in stride time under a dual-task condition of healthy community-dwelling older adults with long-term practice of Dalcroze approach and healthy older adults of similar age without any particular exercise routine	Intervention-control groups, two different tasks	Older women; IG (*N* = 10, aged 79.6 ± 4.9): regular practice of Dalcroze for at least 40 years (once a week, during at least 26 weeks per year); CG (*N* = 11, aged 77.7 ± 4.1): healthy women with no particular exercise routine	Recruited, inclusion criteria: at least 60 years of age, able to walk without an external aid
Treviño and Álvarez-Bermúdez, 2016	Mexico	Evaluate the effect of Dalcroze based intervention among older people	Exploratory, single group design intervention, twice a week for 3 weeks, no control group	Older adults (six women and three men), with a mean age of 69.8, (*N* = 9)	Recruited by the Association La Divina Providencia ABP, a private charity institution in Monterrey, Nuevo León
Treviño and Álvarez-Bermúdez, 2018a	Mexico	Evaluate the efficacy of a Dalcroze based intervention in older adults on the variables related to psychological health, social relationships, affectivity, and state of flow	Exploratory, single group design, twice a week for 4 weeks, no control group	Older adults, women (*N* = 9) and men (*N* = 2), (*N* = 11). Inclusion criteria: (1) ability to walk with no external aid, (2) average cognitive decline according to age, (3) willingness to participate in the study	Recruited
Treviño and Álvarez-Bermúdez, 2018b	Mexico	Evaluate the efficacy of Dalcroze-based intervention on the state of flow among older adults	Quasi-experimental design three experimental groups and a control group	Older adults (*N* = 60)	Recruited through the governmental agency, and a private independent organization for active older adults
Treviño et al., 2018	Mexico	To Investigate the effect of Dalcroze activities on a state of flow	Exploratory, an analytical-type structured questionnaire (closed-ended questions and multiple response alternative)	Older adults (*N* = 41)	Recruited participants from an inpatient 24/7 permanent geriatric residence in the Monterrey metropolitan area
Trombetti et al., 2011	Switzerland	Study whether a 6-month music-based multitask exercise program (i.e., Dalcroze approach) would improve gait and balance and reduce fall risk in community-dwelling older adults at high risk of falling	A randomized controlled trial, music-based multitask exercise program (for 6 months) or a delayed intervention control group (for 6 months)	Community-dwelling older adults at high risk of falling (*N* = 134)	Recruited through multiple strategies, inclusion criteria:(1) age 65 + (2) living in the community, (3) no previous Dalcroze (except in childhood, and (4) at increased risk of falling

**TABLE 3:** Studies of individuals with SEN.

References	Origin	Purpose	Research design/Methods	Target population	Context
Bouloukou et al., 2021	Spain/Greece	Investigate the effectiveness of an interventional Dalcroze/Orff based music-training program (12 week; 40 min.) based on rhythm-perception enhancement in children with dyslexia	Intervention-control groups, quasi experimental design (CG: 16 Greek-speaking students, IG: the remaining 16 (8 females and 8 males)	Students diagnosed with dyslexia, aged 8–9 years, (*N* = 32)	Music training based on multisensory, vocal, acoustic, kinesthetic activities, adapted to the needs of students with dyslexia, within a music course in Greece
Flaugnacco et al., 2015	Italy	Examine the effect of music training (Kodaly and Orff approaches) in enhancing phonological and reading abilities in children with dyslexia	Prospective, multicenter, open randomized controlled trial, consisting of test/ rehabilitation/re-test	Children with a diagnosis of dyslexia, aged 8–11, (*N* = 46)	EdHealth service institution
Habib et al., 2016	France	Investigate the effectiveness of a new method of music intervention (“Cognitivo-Musical Training, CMT”) on reading skills of children with dyslexia	Experimental design, 2 intervention groups (3 days/6 weeks), no control group	Children with severe dyslexia, aged 8–11, (*N* = 12 both groups)	Education: primary school
Kang et al., 2016	USA	Determine whether Dalcroze approach is a feasible intervention for transitional post-acute care BI patients to be used for improving cardiovascular fitness, mobility and cognitive functions	Intervention, twice a week (50 min.) for 6 weeks, no control group	Brain injury patients, male, aged 23–27, (*N* = 6)	Recruited from a rehabilitation hospital
Lakes et al., 2019	USA	Examine qualitative characteristics of and individual responses to a music and movement intervention (Creatively Able) for children with ASD	Pilot intervention for Creatively Able approach, no control group	Children with ASD, aged 7–12, (*N* = 12) study 1, (*N* = 8), study 2	Education: primary school; extra curriculum activities
Sutela et al., 2020	Finland	Identify the development of agency in one student with ASD during a school-year-long Dalcroze-based intervention	Teaching experiment, 21 weekly lessons, during 7 months; no control group	Student with autism, aged 15, (*N* = 1, among 11)	Education: lower secondary, special school
Sutela et al., 2021	Finland	Examine the development of agency in students with special needs during an experiment of classroom music teaching in a special school	teaching experiment, 21 weekly lessons, during 7 months; no control group	See above (based the same intervention)	See above

All interventions were group-based, as is typical for music–movement integration practices (e.g., Juntunen, 2002). Fifteen of the interventions were based or drew on the Dalcroze approach, including three that also integrated other approaches or practices. Three studies used an approach exclusively designed for the intervention (see Table 4). Out of the 18 intervention studies, nine were carried out in Europe (Finland, France, Italy, Poland, Spain, and Switzerland), four in the US, four in Mexico, and one in New Zealand.

**TABLE 4:** The music–movement integration approaches employed.

References	Intervention practice
Fischbacher et al., 2020	In addition to Dalcroze approach, incorporated an additional home exercise program
Bouloukou et al., 2021	Both Dalcroze and Orff approaches
Flaugnacco et al., 2015	Both Kodaly and Orff approaches
Habib et al., 2016	Approach designed by speech therapists for dyslexics
Lakes et al., 2019	*Creatively Able –* approach designed for individuals with ASD.
Choo et al., 2020	*Intuitive Movement Reembodiment* – an original creative dance program designed for older adults with dementia in which the use of music drew on Dalcroze approach.
All the other interventions	Dalcroze (based) approach

The reviewed studies used both qualitative and quantitative data gathering methods. Among the 18 interventions, 13 studies applied only quantitative methods (three of them among those with dyslexia and 10 among older adults), two employed only qualitative methods, and three were mix-methods studies. Four were pilot studies. The research methods/designs are described in Table 5. The sample size was low in most studies, varying from 9–134 participants (Tables 2 and 3).

**TABLE 5:** Research designs of the studies.

Study design	Intervention studies
Qualitative studies (2)	Beaulieu et al., 2017 (action research) Sutela et al., 2020, 2021 (case study)
Mixed methods (3)	Trombetti et al., 2011; Ferguson-Stegall et al., 2017; Choo et al., 2020 (pilot)
Quantitative methods (13):	All the other intervention studies, not mentioned above.
Randomized controlled trials (6)	Trombetti et al., 2011; Hars et al., 2014; Flaugnacco et al., 2015; Lakes et al., 2019 (pilot); Fischbacher et al., 2020 (pilot); Adamczyk et al., 2022
Exploratory (3)	Treviño and Álvarez-Bermúdez, 2016, 2018a; Treviño et al., 2018
Quasi-experimental design (2)	Treviño and Álvarez-Bermúdez, 2018b; Bouloukou et al., 2021
Quasi-experimental design with a control group (3).	Kressig et al., 2005; Adamczyk et al., 2020, 2022

The measures used to assess the outcomes were selected according to the target group and research aims. The studies on older adults used measures typical in geriatrics and psychology, and the studies on individuals with dyslexia applied those typical in speech therapy, and so on. In all studies, the measures employed were carefully chosen and clearly detailed. Most interventions had pre- and post-testing.

### Interventions and outcomes

In this result section, we will first answer the main research question: What were the outcomes of the music–movement integration interventions among vulnerable groups?

#### Individuals with varying SEN or impairment

Studies among individuals with SEN showed evidence of the effectiveness of music–movement integration in supporting participation, experiential music learning, and agency. Interventions among those with dyslexia improved participants’ specific linguistic skills. The studies, which will be summarized below, emphasized the potential of music–movement integration as an inclusive and multisensory practice.

A school-year-long intervention by Sutela in a music classroom of a special comprehensive school was reported in two articles (Sutela et al., 2020, 2021). According to the latter work, Dalcroze-based music–movement integration supported the agency of 15-year-old students with SEN as they became recognized by others as active music makers and enhanced their initiative, decision-making, interaction, engagement, and expression of emotions. The authors described how, as the intervention proceeded, hyperactive students gradually increased their self-regulation, which then enabled them to concentrate better on interactions with peers and take initiative. Sutela et al. (2020) reported the development of agency of one student with ASD: The student’s compulsive and other behaviors typical of this condition decreased, and he transformed from a passive outsider into an active participant. This was observed not only by the researcher and teacher but also by school staff and his peers outside the classroom.

Similar results were seen in two pilot studies reported in Lakes et al. (2019), wherein 20 children with ASD participated in a music–movement intervention specially designed for the target group (Creatively Able). The results showed some group-level reductions in compulsive and other behaviors commonly seen in those with ASD. However, there were notable individual differences in how children responded to the intervention. These studies suggest that music–movement integration provides an alternative (multimodal) method of teaching and communication through movement, gestures, and music instead of verbal communication.

Three of the reviewed studies involved children with dyslexia (Flaugnacco et al., 2015; Habib et al., 2016; Bouloukou et al., 2021). The interventions focused on musical training including movement activities and were based on the similarities between music and language.^4^ All the studies reported positive changes and improvements in specific linguistic skills, either for individual progress or compared to control group participants. The progress often took place along with advancement in rhythmical and metrical abilities, which were related to improvements in auditory processing, prosodic and phonemic sensitivity sequencing abilities, as well as auditory and temporal orienting of attention. The findings strongly imply the beneficial influence of music training on phonological awareness and reading skills through a specific effect on perceptual and cognitive abilities shared by music and language. All these studies argue for using music/music–movement as part of systematic therapeutic and pedagogical practices for children with dyslexia. In the following, we discuss each study in more detail.

The study of Habib et al. (2016) examined the effectiveness of the cognitivo–musical training (CMT) method, which was designed by speech therapists. The intervention consists of musical exercises that activate “jointly and simultaneously sensory (visual, auditory, and somatosensory) and motor systems, with special emphasis on rhythmic perception and production” (p. 1). Two experiments (considered here as one intervention) were conducted among two different samples of children with dyslexia, one in specialized classes and the other in a primary school-like environment. Both experiments resulted in significant improvements in some untrained, linguistic, and non-linguistic skills, such as “in categorical perception and auditory perception of temporal components of speech” and “auditory attention, phonological awareness (syllable fusion), reading abilities, and repetition of pseudo-words” (p. 1). Importantly, most improvements lasted over a six-week period without training. These studies suggest there are benefits to using multisensory and multimodal music training in systematic therapeutic and pedagogical practices for children with dyslexia.

Flaugnacco et al. (2015) examined the effectiveness of music training in developing reading and phonological abilities. Children with dyslexia (but without language or comorbid attention disorders) participated in training sessions for seven months. Movement was part of the music training influenced by Kodaly and Orff approaches. Those with music training clearly improved their reading accuracy, both in text reading and pseudo-word reading. Rhythmic reproduction (not working memory or auditory attention) and the ability to determine metrical structure predicted phonological awareness, and improvement in rhythmic reproduction predicted improvement in phonological abilities. These findings imply a causal relationship between rhythm-based processing and language acquisition and phonological development.

The study of Bouloukou et al. (2021) investigated the influence of a music-training program focusing on the perception of rhythm among those with dyslexia. The 12-week intervention was conducted with primary school students. The study showed improvement in students’ performance related to rhythm reproduction, visual sequences, word recognition, grammar, and spelling; participants in the control group did not improve in terms of their verbal or written comprehension, verbal expression, or completion of sentences. The research findings offer evidence that incorporating properly modified music training into the primary school curriculum and continuous music education elsewhere may have positive effects on children with dyslexia.

Among brain injury patients, the study of Kang et al. (2016) examined the influence of music–movement integration on cardiovascular function, cognitive function, and balance. Due to the small sample size, statistically significant differences were not identified between the pre- and post-tests. However, the results indicated improvement in the abovementioned measures. The only participant who completed the exit survey strongly agreed with the benefits of the Dalcroze approach in rehabilitation and enjoyment. Similarly, enjoyment of music and social interactions were reported by the researchers. Feedback was positive overall.

#### Older adults with a risk of falling or a decline in health

In this section, we report nine studies based on interventions conducted among older adults (65 +) facing the risk of falling or the threat of a decline in their health and/or physical strength because of age and/or living conditions. All the interventions were Dalcroze-based. In short, all the studies reported positive outcomes, including reduced rates and risk of falling; improved accuracy and speed of movements, postural stability, gait variability, and balance, both in single-and dual-task conditions; an enhanced state of flow,^5^ sense of control, and enjoyment; and decreased self-consciousness.

Based on the findings of Beauchet et al. (2003) showing that variation in gait and speed increases notably in older adults when they engage in a counting activity, Kressig et al. (2005) examined a group of women who had engaged in regular Dalcroze practice for at least 40 years to determine whether this type of long-term music–movement intervention would make a difference in gait. The study showed that long-term Dalcroze practice can prevent age-related increases in stride-to-stride variability when dual-tasking (counting backward while walking). In contrast, gait variability increased considerably in the healthy older adults in the control group when dual-tasking. These results were the first to demonstrate such positive influences of Dalcroze practice in older adults.

The study by Trombetti et al. (2011) examined the influence of a six-month Dalcroze program with multitask exercises on the gait, balance, and functional performance of older adults living in a community who had a risk of falling and no previous experience with such activities. The participants’ gait variability and balance improved in a statistically significant way, and the rates and risk of falling declined. Additionally, gait performance under the dual task improved, and stride length variability decreased also when the speed of walking was varied.

After three years, Hars et al. (2014) conducted a follow-up study among those who had maintained exercise program participation (long-term intervention group) and among those who had discontinued participation (control group). In four years, the intervention participants’ gait and balance (one-legged stance time) improved significantly. They also did better on the timed-up-and-go, five-times-sit-to-stand, and handgrip strength tests than those in the control group. The results suggest that a long-term music program with multitask exercises is promising in preventing age-related physical decline in older individuals and that physical exercises aimed at preventing falls are effective in the long term.

A similar study was conducted by Ferguson-Stegall et al. (2017) to examine whether a three-month Dalcroze intervention would improve gait and balance, self-perceptions of health, and fear of falling in older adults living in a community. The participants’ gait speed improved significantly, including when dual-tasking. The authors recommended using the Dalcroze approach in programs for older adults to reduce the risk of falling.

Adamczyk et al. (2020) studied the effects of Dalcroze practice on dynamic agility under single- and dual-task conditions in women who were over 65 years old and lived in a community (in Poland). After the 12-week program intervention, the participants scored significantly better on both single- and dual-task tests than those in the control group. Based on the same intervention, Adamczyk et al. (2022) investigated the influence of the program on postural stability. The intervention participants’ accuracy and the speed of their movements improved significantly compared to those of the members of control group. The study concluded that using the Dalcroze approach may improve postural stability by increasing the speed and accuracy of body (torso) movements.

The pilot study of Beaulieu et al. (2017) was performed among older adults in a community health program based on the Dalcroze approach. The study used focus group interviews to examine participants’ experiences related to the strengths and possible challenges of the program. The identified strengths involved: “(1) social and physical pleasure; (2) improved health, including balance, gait, recovery after an injury, confidence in mobility, metamemory skills, and a greater understanding of health promotion and fall prevention strategies; and (3) the collaborative nature of individualized support” (Beaulieu et al., 2017, p. 276). The identified challenges by the participants related to transportation, schedules, and participants’ varying skills.

The exploratory study of Treviño and Álvarez-Bermúdez (2016), which had a single-group design, assessed the effect of a six-week (twice a week) Dalcroze intervention on the “state of flow” in older adults (mean age 69.8) with a pre- and post-questionnaire. Although the participants had limited musical and physical skills to engage in the activities, they were motivated and enjoyed participating. The perceived challenges of the exercises correlated with the participants’ level of musical and physical abilities. Toward the end of the intervention, the participants began to feel more comfortable with the exercises and more in control of the situation.

Based on the study described above, Treviño and Álvarez-Bermúdez (2018b) evaluated the effectiveness of an eight-week (once a week) Dalcroze intervention on the state of flow in older adults. The participants lived in a vulnerable socio-demographic area of the Monterrey metropolitan area. The authors reported that the intervention partially promoted a state of flow in the intervention participants and had a significant impact on the autotelic experience in one group. The need for longitudinal investigation was also recognized.

A similar study (Treviño et al., 2018) was conducted among older adults living in a geriatric residence in Monterrey investigating the impact of a 10-week Dalcroze intervention on state of flow. The participants’ sense of control improved, and they became less self-conscious; these results were statistically significant. The intervention also enhanced the state of flow in the sample. With the increasing number of older adults worldwide, the Dalcroze approach can be considered a valuable option for an ecological and non-pharmacological intervention for older people in public health systems.

#### Older adults with cognitive decline or impairment

Three of the reviewed studies were conducted on older adults with some cognitive decline, impairment, or dementia. Two studies (Treviño and Álvarez-Bermúdez, 2018a; Fischbacher et al., 2020) applied the Dalcroze approach, and one was based on the specially designed intuitive movement re-embodiment (IMR) program in which the music component drew on the Dalcroze approach and the creative movement incorporated ideas from the Laban approach (Choo et al., 2020). The studies reported improvement in positive affectivity, state of flow, quality of life, sense of humor, imagination, motivation for dance, and joyful interaction. However, one of the studies did not obtain any results because of low attendance.

First, the study of Treviño and Álvarez-Bermúdez (2018a) evaluated the effectiveness of a four-week (twice a week) Dalcroze intervention among older adults on “psychological health, social relationships, affectivity, and the state of flow” (p. 23). The inclusion criteria included some age-related cognitive decline and the ability to walk with no external aid. The intervention had positive effects on the variables addressed in the study, especially on positive affectivity and state of flow.

The 12-month pilot study of Fischbacher et al. (2020) examined the safety, feasibility, and effectiveness of a Dalcroze-based program and a home exercise program (designed to prevent falls) among older adults with mild cognitive impairment or early dementia. The study showed no differences between the intervention and control groups. The low adherence and small sample size were challenging for the study, which noted the difficulties involved in the recruitment of older adults with mild cognitive impairment or dementia for a long-term intervention.

In the study of Choo et al. (2020) older adults with dementia, who were grouped according to the degree of their dementia, participated in 10 weekly sessions. The data included self-reported ratings of quality of life and qualitative onsite observations. There was a statistically significant improvement in quality of life after the 6th session. According to the qualitative analysis, the program improved sense of humor and imagination as well as motivated the participants to dance and interact with joy.

### Outcome measures employed

The reviewed studies used both qualitative and quantitative data gathering methods. The selection of measures was based on the target group and the research aims, resulting in questions of validity. While the studies among older adults employed measures typically used in geriatrics and psychology, the studies on those with dyslexia applied those typical in speech therapy, and so on. For all the studies, the measures were carefully chosen and clearly articulated, with most interventions involving both pre- and post-testing.

#### Individuals with special needs

When studying the benefits for students with SEN, both qualitative and quantitative research methods were utilized (see Table 5). Video analysis was used in three studies (Lakes et al., 2019; see Nvivo in Sutela et al., 2020, 2021). In the study of Lakes et al. (2019), participants were also rated using the Response to Challenge Scale, which aids the observer in rating a child’s self-regulation. Individual outcomes in turn were analyzed using the statistical analysis system with the linear mixed-model procedure.

To assess language skills (reading, writing) among individuals with dyslexia, several types of tests were applied. For example, in Habib et al. (2016), “three tasks tapping into different aspects of auditory and speech perception were used: categorical perception (identification and discrimination tests), syllabic duration and pitch variations” (p. 4). In the study of Flaugnacco et al. (2015), rhythm reproduction and tapping tasks were used to assess, for example, accuracy and speed of reading words and text, phonological knowledge, auditory attention, verbal short-term and working memory, as well as temporal processing. Bouloukou et al. (2021) employed the LAMDA test to evaluate the learning skills of students.

#### Older adults

To measure the outcomes in older participants, several measures typical in geriatrics were applied. For example, gait and balance were assessed using an electronic pressure-sensitive walkway and angular velocity transducers (Kressig et al., 2005; Trombetti et al., 2011). Balance and coordinated stability were measured with the swaymeter test (Ferguson-Stegall et al., 2017). Measuring dual-task ability incorporated counting backwards while walking at a self-selected speed (Ferguson-Stegall et al., 2017). In addition to physical examination, Trombetti et al. (2011) used functional tests, instrumental gait and balance analysis, and interviews to collect data about “sociodemographic characteristics, fall history, nutritional status, physical activity level, and neuropsychological status” (p. 526). Similar measures were applied in the follow-up study by Hars et al. (2014). As completing measures, Ferguson-Stegall et al. (2017) used questionnaires to assess participants’ perceptions of their overall, physical, and mental health. Fear of falling was determined with the Tinetti Falls Efficacy Scale.

In the study of Adamczyk et al. (2020), dynamic agility was ascertained by the timed-up-and-go test, which was conducted both in single- and dual-task conditions (walking and simultaneously counting down from 60 every third step). Adamczyk et al. (2022) also used a test on the AMTI AccuSway Plus posturography platform, together with Balance Trainer software, to determine postural stability.

Treviño and Álvarez-Bermúdez (2016, 2018b,a; also Treviño et al., 2018) applied a wide variety of tests and other measurements from the field of psychology. For example, for assessing the state of flow, flow state scales were used, and the level of enjoyment of physical activity was assessed according to the physical activity enjoyment scale. Other measurement instruments included the subscales of the domains of psychological health and personal relationships of the reduced version of the World Health Organization Quality of Life Scale and Positive and Negative Affectivity Scale with a total of 20 items.

Only one study applied qualitative data alone: Beaulieu et al. (2017) used focus group interviews to examine older adults’ perceptions of the strengths and challenges of the constructed program and their recommendations for improvement. Choo et al. (2020) employed a mix-method design, including self-reported ratings of quality of life with the World Health Organization’s questionnaire, alongside qualitative onsite observations.

### Intervention exercises

#### Individuals with special needs

Among studies on individuals with SEN, three interventions (out of six) were at least partly based on the Dalcroze approach (see Table 4; Supplementary Appendix 1). The others were based on Kodaly and Orff approaches or methods designed for the specific group of individuals. In older adults, all interventions applied the Dalcroze approach in some way. For instance, in the creative dance program of Choo et al. (2020) designed for older adults with dementia, Dalcroze ideas guided the use of music.

As Dalcroze practice includes a variety of possible focuses and exercises (see e.g., Juntunen, 2016), the selection of exercises reflected the target group and aims of the intervention: the interventions were adapted to the needs of each target group and addressed the examined variables. For example, in the study of Habib et al. (2016) on those with dyslexia, the intervention was designed by speech therapists and built on an understanding of what constitutes an effective intervention for such individuals. Thus, the exercises focused on both multisensory and multimodal engagement and required transcoding processes from one modality to another. Furthermore, visuo-spatial and motor engagement and building connections between music and language were used. In their intervention for older adults with dementia, Choo et al. (2020) employed a dance program that aimed to promote better quality of life for people with dementia by providing memory stimulation, mood moderation, and social interaction through music–movement integration. In the study of Sutela et al. (2021) examining students’ manifestation and development of agency, the exercises enabled the students with SEN to practice and develop their motor and movement skills, communication, and autonomy. Therefore, the exercises included lead and follow, moving to music, rhythmic movement, and quick response. In particular, lead and follow and creative exercises were designed to offer the students an opportunity to take initiative and express their agency.

In the study of Kang et al. (2016) examining the possibilities of the Dalcroze approach for brain injury patients’ cardiovascular fitness, mobility and cognitive functions were examined through activities such as walking in tempo, stopping as cued to the music, manipulating props, and creative freeform movements. Memory and quick reaction games were also utilized to help the participants achieve the desired outcomes.

While some studies described the intervention exercises in detail (see Treviño and Álvarez-Bermúdez, 2016, 2018a,b; Treviño et al., 2018), others focused on the main features and only provided a general description of the exercises. For example, Kressig et al. (2005) stated that their intervention consisted of “multitask exercises, mostly performed to the rhythm of improvised piano music” (p. 728). As in the interventions with those with dyslexia, movement as part of music training was often applied to express and experience rhythmic elements of music, such as pulse, meter, or time values, and integrated with speech.

As using music with individuals with autism is supposed to both eliminate potential distractions and over-stimulation and support movements, specific requirements for the music were presented. These included employing music with an easy-to-follow and steady rhythm (Lakes et al., 2019; Sutela et al., 2020), with a melodic structure that does not over-stimulate hypersensitivity (Lakes et al., 2019), and without lyrics (Lakes et al., 2019). A steady rhythm offered structure and organization to the sessions and movements (Lakes et al., 2019). The exercises were mostly performed in pairs or in a group, which supported student interaction and helped build social skills (Lakes et al., 2019; Sutela et al., 2020). Different objects, such as tennis balls, were used to make the perception of musical elements more concrete (e.g., students bounced the balls in tempo or rolled them according to a certain meter).

For children with dyslexia, Habib et al. (2016) employed specially designed CMT that focused on the rhythmic and temporal aspects of music training. When using Kodaly and Orff approaches, Flaugnacco et al. (2015) made use of activities selected from training books and programs and adapted them to focus on rhythm and temporal processing. Percussion instruments, rhythm syllables, rhythmic body movements accompanying music, and sensorimotor synchronization games were employed. In the intervention in Bouloukou et al. (2021), “the music training was based on innovative multisensory, vocal, acoustic, kinesthetic activities, adapted to the needs of students with dyslexia” (p. 458). The exercises included adapted speech, movement, touch, hearing, and pulse sensing activities with participants to develop their perception of the musical rhythm (see Supplementary Appendix 1).

#### Older adults

Of the 12 interventions among older adults, 11 were based on the Dalcroze approach. The activities consisted of Dalcroze exercises in which the participants expressed and responded to (improvised piano) music through (synchronized and improvised) body movements, including a variety of multitask exercises. Again, the exercises reflected the aims of the study. For example, in studies that examined gait and balance in dual-task conditions, the exercises challenged gait and balance but also functions of memory, attention, and coordination (Beauchet et al., 2003; Trombetti et al., 2011; Hars et al., 2014; Adamczyk et al., 2020, 2022) to integrate motor and cognitive functions simultaneously.

During a session, exercises gradually became more complex. For example, they started as single-task and then turned into multitask exercises. In some cases, exercises involved the handling of objects (such as percussion instruments or balls) (e.g., Trombetti et al., 2011). Basic exercises were comprised of walking (in time) with music and responding to its rhythmical changes. Exercises applied a wide range of movements and challenged balance by requiring multidirectional weight shifting, sequences of walking and turning, and exaggerated upper body movements (while walking or standing).

Dalcroze-based exercises could involve different rhythmic themes, such as double and triple speed or slowing down, rhythmic transformation, and polyrhythms (Adamczyk et al., 2020). In addition, “inhibition and stimulation of movement, exercises reflecting dynamic, agogic, and articulatory courses in music, improvisation of movements, and exercises shaping independence of movements and their coordination were performed” (Adamczyk et al., 2020, p. 2; see also Adamczyk et al., 2022). The interventions of Treviño and Álvarez-Bermúdez (2016, 2018a,b) and Treviño et al. (2018) applied the Dalcroze approach in carefully timed, structured, and sequenced sessions (Supplementary Appendix 2).

Choo et al. (2020) presented an original creative dance program designed as a community dance activity for people with dementia. It used music and natural gestures to construct a series of dance exercises. After the teaching period, the participants were guided to perform the practiced routine independently with the same music that included a variation. The program aimed to enhance the quality of life of people with dementia by providing memory stimulation, mood moderation, and social interaction.

### Effectiveness of music–movement integration

In this section, we will answer our fourth research question: What qualities of music–movement integration were identified as effective? The main effective qualities included (1) the mutually supportive potential of music and movement, (2) rhythmic performance and experience, (3) active engagement and a related state of flow, joyfulness, and other positive experiences, (4) integration of motor and cognitive faculties, as in dual-task exercises, (5) multisensory integration and multimodal expression, and (6) non-verbal communication and interaction.

First, most studies were based on the mutually supportive potential of music–movement integration wherein music supported movement, movement supported listening, and the two together resulted in animation and participation. Movement also enabled bodily engagement and working with others. Thus, in addition to sensory integration, this working method offered motivating and rewarding social engagement with others. Working with live music was considered extremely valuable as it supported participants’ movement in terms of style and rhythm, and listening to it was experientially satisfying. In some music–movement integration interventions, either music or movement was considered primary. For example, among those with dyslexia, the studies emphasized the possibilities of music as primary whereas among individuals with autism, movement seemed to be more important as it allowed active participation and supported agency.

In many reviewed studies, the positive influence of music–movement activities was principally identified as related to *rhythm*, especially in those focusing on dyslexia. For example, according to Flaugnacco et al. (2015) “the ability to detect the temporal regularity of an auditory sequence” (p. 12/17) in musical exercises strongly correlated with phonological skills. Their findings “highlight an important role of rhythm on phonological perception and production” (p. 13/17), in line with the results other studies (see e.g., Huss et al., 2011; Reifinger, 2019). In many Dalcroze-based interventions, it has been emphasized that the exercises were done to the rhythm of (improvised) music (e.g., Beauchet et al., 2003).

Studies that focused on mental health among older adults particularly underlined *active and social engagement and positive experience* as important outcomes of participation in music–movement activities (e.g., Treviño and Álvarez-Bermúdez, 2018b). Flaugnacco et al. (2015) noted that the emotionally engaging and playful nature of the exercises motivated children to participate in and value the training. Trombetti et al. (2011) stated that Dalcroze activities seem “to be able to change patterns of physical activity in elderly people by providing a strong motivation for the initiation and maintenance of exercise behavior, especially in women…who are often less physically active than men” (p. 531). Over half the participants entered the program again after the study at their own cost.

One identified strength of music–movement integration was that it stimulated both motor and cognitive functions and thus was particularly beneficial for cognitively impaired older adults (Fischbacher et al., 2020). The studies on older adults addressing (dual-task) gait variability (e.g., Kressig et al., 2005; Trombetti et al., 2011; Ferguson-Stegall et al., 2017) or quality of life of older adults with dementia (Choo et al., 2020) viewed the strength of music–movement training to lie in *dual- or multitask exercises*, forcing participants to constantly focus, perceive, and respond accordingly. When exercises include both cognitive and physical tasks at the same time, it is postulated that they lead to better results compared to performing tasks separately (Adamczyk et al., 2022). For this reason, researchers have found Dalcroze practice to be particularly efficient in comparison with other attention-demanding practices, such as Tai Chi. According to Trombetti et al. (2011), the possible strength of these exercises for improving gait safety, balance, and functional capacities of older adults was related to “automated tasks, to task coordination skills development, or to both” and to improving attention and executive function (p. 530).

Other strengths of music–movement integration identified in studies on individuals with special needs, such as ASD, included multisensory activities, experiences, and learning (integrating visual, auditory, kinesthetic, and tactile senses) and multimodal expression (e.g., Sutela et al., 2020). Several studies suggested that music–movement integration provided an alternative to verbal communication and interaction through embodied interaction via movement, gestures, and music, thereby promoting inclusion, especially among students for whom verbal communication was challenging (such as those with ASD) (Sutela et al., 2020, 2021). Music–movement integration also enabled the processing and expression of emotions on a bodily level, without conscious reflection and words.

## Discussion

Our literature review shows that older adults and individuals with SEN are the groups primarily addressed in Dalcroze-based intervention studies outside of more conventional music and arts education contexts. Each reviewed intervention study was targeted at a specific group. Accordingly, the intervention exercises and associated research concentrated on the key issues and challenges for the target group. Hence, studies among older adults addressed gait, balance, dual-tasking, active participation and rehabilitation, confidence in mobility, metamemory skills, advanced understanding of fall prevention strategies, enjoyment, and wellbeing as well as applied methods typical in geriatrics and psychology.

Two out of three interventions were carried out among older adults, which suggests that Dalcroze based activities are increasingly being used among this population. This may be due to their various researched and recognized possibilities to support physical and cognitive faculties, including balance and multi-tasking (see e.g., Schmitt and Kressig, 2008). Older adults’ mobility strongly correlates with quality of life while falls often restrict mobility, cause a decline in daily activities, and increase the risk of institutionalization. Therefore, preventing falls in older adults has become an important issue. High stride-to-stride variability and the inability to perform multiple tasks at the same time, such as talking while walking, have been considered potent predictors of falls (Beauchet et al., 2003). Measures to reduce falls include improving balance and multitasking skills. Other challenges with older adults include their tendency to passivity, boredom, loneliness, and isolation, which necessitates looking for ways to engage older adults with different types of motivating and rewarding activities.

In studies among special needs students, the focus of the interventions was on inclusion, reductions in stereotyped and compulsive behaviors, self-regulation, learning skills, interaction and engagement; in those with dyslexia, rhythmic and metrical abilities, auditory attention, phonological awareness, and linguistic skills were of particular concern; and among brain injury patients, the concentration was on cardiovascular function, perceptual and cognitive abilities and functions, rehabilitation, and enjoyment.

Most interventions (15/18) applied Dalcroze-based exercises in interventions. The interventions included a variety of exercises in which music and movement were reciprocal: movement reflected music and supported music listening while music supported movement and motivation to participate. Three studies on those with dyslexia did not mention Dalcroze but still used rhythmic movement with music. Lakes et al. (2019) applied a novel approach designed for children with ASD, and Choo et al. (2020) used an approach designed for people with dementia for quality-of-life improvement. In addition to the abovementioned aspects—the reciprocal and positive effects of music and movement on each other, physical performance, and experience—the identified beneficial qualities of music–movement integration (RQ 4) included an inclusive learning environment; speech, language, and musical proficiency; social interaction and communication; and the integration of cognitive functions, emotions, multisensory experiences, and multimodal expression. Similar strengths of music–movement integration have been identified in other studies (e.g., Zatorre et al., 2007; Donnellan et al., 2012; Altenmüller and Scholz, 2016; Juntunen, 2020a,b).

The positive findings of the reviewed studies are supported by previous research investigating practitioners’ experiences of the effectiveness of music–movement integration, focusing especially on multisensory integration and multimodality. Regarding individuals with sensory impairments, Bang (2009) argued that in children with hearing loss, music–movement integration supports the development of their movement skills, awareness of body functions, and linguistic skills, in addition to providing the enjoyment of interacting with others (see also Salmon, 2010). According to Pino et al. (2022), music–movement integration, especially by integrating sound and the usage of body and space in learning, offers a wide range of opportunities for multisensory learning and facilitates participation and inclusion for students with visual disabilities (see also Coleman, 2017). Nelson and Hourigan (2016), who have used multisensory methods in supporting professional musicians with dyslexia, suggested that the integration of tactile (touch), kinesthetic (body movement), aural (hearing), and visual (sight) senses can be beneficial when learning to read music.

Many reviewed studies concluded that the intervention would have needed more time or a longer period for stronger evidence. Indeed, longitudinal studies are required to reach desired outcomes (Kang et al., 2016; Sutela et al., 2021) or to confirm the identified outcomes and benefits (e.g., Treviño and Álvarez-Bermúdez, 2018a,b). Yet, it is challenging to engage participants (also personnel) for long interventions (e.g., Fischbacher et al., 2020). Kang et al. (2016) also reported the challenges of group-based implementation intervention in a clinical setting. Low participation (if voluntary) and small groups present challenges even in shorter interventions; however, interventions are difficult to implement with a large sample.

To our knowledge, our systematic review is the first to address music–movement integration interventions among vulnerable individuals. It is also the first review to look at the actual exercises and highlight the beneficial elements of music–movement integration identified in the studies. The first task was to present our definition of the term “vulnerable” and to map which groups of individuals meeting the definition had been addressed in previous studies. As we wanted to have a comprehensive understanding of prior research, we included all the studies that met the inclusion criteria, i.e., that addressed either individuals with SEN or older adults. This delimitation of studies can be considered both a positive choice and shortcoming of the study. Focusing on only one vulnerable group (e.g., older adults) would have made the review more concise but would not have enabled a broad scope. In future studies, focusing on one group could be considered. However, we did not know at the outset which vulnerable groups the reviewed interventions would potentially address. Indeed, it was surprising that two thirds related to older adults. It seems there is a growing need to support the health and wellbeing of older adults whose number is increasing globally (Schmitt and Kressig, 2008). Another critical moment in the study was to define and formulate the inclusion criteria for music–movement integration. We decided to focus on Dalcroze-based practices following the arguments set out earlier in the text.

## Conclusion

The main objective of our systematic review was to synthesize previous intervention studies published from January 2000–October 2022 to evaluate the effectiveness of Dalcroze-based or similar music–movement integration in a variety of contexts for vulnerable groups. Most reviewed studies (16/18) reported beneficial outcomes. The study of Kang et al. (2016) did not obtain statistically significant results, but the findings indicated improvement in the examined variables. The only intervention without any outcomes was the study by Fischbacher et al. (2020), in which the final number of participants and adherence remained low.

The results were in line with the goals and hypotheses set for each study. However, as studies in each field had different goals, used different controlled variables, and created explanations of one or successive experiments using field-specific vocabulary, assumptions, and interpretations, it was challenging to make an overall evaluation of the effectiveness of music–movement integration. As Gallagher (2017) argued, “each science tends to develop its theoretical bases on its own particular assumptions, in its own vocabulary, and often in isolation from the insights of other sciences” (p. 22). In sum, the results reflect what has been suggested within enactivism in the cognitive sciences, that is that the body influences cognition in terms of at least three dimensions, namely “sensory-motor contingencies, affective factors, and intersubjective processes” (Gallagher, 2017, p. 42). The role of music can be viewed as supporting movement, enlivening the experience, and enhancing motivation. Moving with music promotes multisensory-motor integration, generates positive emotions, and enhances brain network integration (Shanker, 2012).

To measure the outcomes of the interventions, quantitative, qualitative, and mixed methods were used. The choice of measures reflected the field of each target group. Thus, among studies on older adults, for example, the measures were those relevant to and credible in geriatrics. Additionally, participants’ experiences were considered relevant in studies among older adults and students with SEN.

In this study, we highlighted the potential and effectiveness of music–movement integration among vulnerable groups. The reviewed studies suggested a variety of practical implications and applications for their findings. Indeed, as the number of older adults and people with SEN is increasing, the holistic functional and inclusive benefits of music–movement integration, which were highlighted in the reviewed studies, should be carefully considered in a variety of contexts in education, therapy, and rehabilitation. We believe that our review for its part offers knowledge and confidence to educators, therapists, and medical personnel for using music–movement integration in their work in schools, senior centers, and other institutions and practices. Yet, further studies on the effectiveness of music–movement integration designed for different vulnerable groups are needed. It is crucial to examine, for example, how the identified benefits of music–movement intervention translate into real life. In addition, we recommend that future studies carefully describe the intervention practices and exercises used in order to examine and consider the effectiveness of the exercise types in more detail. This would assist researchers and practitioners in better understanding the key factors and possibilities of music–movement integration in relation to different areas of development and the capabilities of (vulnerable) individuals.

## Author contributions

M-LJ has had the main responsibility for the preparation, revision, and editing of the text. Both authors have been equally engaged in the conceptualisation, methodology, resources, data search, data analysis, and Supplementary material.

## Footnotes

1 Another example of a multisensory process would be the integration of visual and auditory information in the perception of speech.

2 Because of this, most studies of dance were excluded. However, we did not consider this to be problematic since the effectiveness of dance among vulnerable individuals has been examined in many previous literature reviews. For example, for the effectiveness of dance among individuals with ASD, see Koehne et al. (2016) and DeJesus et al. (2020); among older adults, see Keogh et al. (2009) and Meng et al. (2020); among children with disabilities, see May et al. (2021); and among individuals with Parkinson disease, see Earhart (2009). For promoting health and wellbeing, see Sheppard and Broughton (2020). Furthermore, in dance, the relationship between music and movement is different from how we perceived it in this study.

3 The flow diagram is created based on the flowchart offered by Zen Flowchart (https://www.zenflowchart.com/guides/prisma-flowchart) and is published with permission.

4 The intervention was built on the hypotheses that (1) music training improves the brain circuits shared by music and language processes; (2) the temporal and rhythmic elements of music positively affect multiple dimensions of the “temporal deficit” characteristic of certain types of dyslexia; and (3) cross-modal integration in exercises is useful (Habib et al., 2016, p. 1).

5 As described by psychologist Mihhály Csíkszentmihályi, state of flow is reached when a person becomes fully immersed in an activity.
