# Supplementary material for: The effectiveness of music–movement integration for vulnerable groups: a systematic literature review
Source: Front Psychol. 2023 Aug 31;14:1127654. doi: 10.3389/fpsyg.2023.1127654 (PMC10513045; doi:10.3389/fpsyg.2023.1127654)
Supplement: Supplementary file 1 [file Data_Sheet_1.docx]

Appendix 1. Examples of music-movement intervention activities and exercises.

The exercises are presented according to the participant group.

*Individuals with variety of special needs*

1. Lead-follow exercises, where students take initiatives and create gestures and movements to express themselves (Sutela et al., 2021)
2. Start/stop or quick reaction exercises (Kang et al., 2016; Sutela et al., 2021)
3. Learning a song with the movement (Sutela et al., 2021)
4. Beat synchronization (Kang et al., 2016; Sutela et al., 2021)
5. Dance (Sutela et al., 2021)
6. Memory games (Kang et al., 2016)
7. Imitation of movement or mirroring (Kang et al., 2016; Sutela et al., 2021)

*Individuals with autism*

1) Mirroring activities in synchrony or imitation (Lakes et al., 2019; Sutela et al., 2020)

2) Creative movements and improvisation (Lakes et al., 2019; Sutela et al., 2020)

3) Developing a story through movement (Lakes et al., 2019)

4) Songs with movement (Sutela et al., 2020)

5) Rhymes and rhythmic cues (Sutela et al., 2020)

6) Rhythmic movement activities like hopping, marching, dancing, or body percussions (Sutela et al., 2020)

7) Use of different objects/materials to study tempo or meter (Sutela et al., 2020)

*Individuals with dyslexia*

Example 1:

Exercises used in Cognitivo-Musical Training (see Habib et al., 2016) that focuses on the rhythmic and temporal aspects of music training included:

1) Exercises concerning, for example, pitch, duration, tempo, pulsation, and rhythm of music and aiming at developing both the perception and the production of music. The exercises incorporated body movements in line with the music.

2) Percussion and rhythmic bodily exercises (practiced with a psychomotor therapist)

3) "A series of musical exercises involving jointly and simultaneously sensory (visual, auditory, somatosensory) and motor systems (with special emphasis on rhythmic perception and production), engaging the child into transcoding processes from one modality to another.”

4) The connection between music and language was supported through exercises that involved both speech and music (such as nursery rhymes, tracing the prosody of a sentence on a sheet of paper). (Habib et al., 2016, p. 3).

Example 2:

Dalcroze and Orff based intervention, designed for individuals with dyslexia (Boukolou et al. 2015, p. 458),

including rhythmic exercises based on innovative multisensory, vocal, acoustic, and kinesthetic activities. The activities adapted speech, movement, touch, hearing, and pulse sensing, aiming at improving as many skills as possible, such as rhythmic perception, memory, attention, and concentration as well as the auditorial ability, motor ability and the visual-spatial ability. The activities were designed to be enjoyable, without causing stress to students.

Sample exercises:

• The teacher keeps the tempo of a song steady and asks each student individually to walk in rhythm while rhythmically bouncing a ball on the floor.

• The teacher hides the metronome in the classroom and the students search for the metronome by sound and clap to the rhythm at the same time.

• The teacher creates the path using large cards with musical time values, such as quarter and eight notes, drawn on them. Students are asked to walk along the path while striking the percussion instrument according to the time values drawn on each card.

• The students imitate the shapes of note values using their bodies, upright or on the floor.
