# Supplementary material for: The effectiveness of music–movement integration for vulnerable groups: a systematic literature review
Source: Front Psychol. 2023 Aug 31;14:1127654. doi: 10.3389/fpsyg.2023.1127654 (PMC10513045; doi:10.3389/fpsyg.2023.1127654)
Supplement: Supplementary file 2 [file Data_Sheet_2.docx]

Appendix 2. Dalcroze-based exercises among older adults

The intervention of Adamczyk et al. (2022, p. 3/9):

Each class consisted of three parts: warm-up (~ 10 min), main part (~ 30 min) and cool down (~ 5 min). Dalcroze exercises consisted mainly of  the repetition of present musical sequences using body movements. In addition to the physical layer, the cognitive layer was equally important: while performing a given movement exercise, participants had to focus in order to react appropriately to additional tasks, such as changes in sound pitch of the piece (e.g. while pitch was high they had to walk on their toes, when low—in a half squat) as well as changes of dynamic, agogic and articulatory sequences in music. Exercises included movements based on rhythmic themes where participants had to adjust the speed of their movement to the tempo of the music, rhythmic transformation of themes and polyrhythms (the arms move in a different rhythm than the legs). There were also inhibition-incitation exercises, which is described to imply stopping moving when the music stops and resuming movement when the accompaniment starts again, improvisation of movements and exercises developing control of body balance, independence of movements and their coordination.

The interventions of Treviño and Álvarez Bermúdez (2016, 2018a,b) and Treviño et al. (2018) applied the Dalcroze approach in carefully timed, structured and sequenced sessions, which included

(1)  Movement warm-up, with and without music

(2)  Rhythmic movement exercises, including leading-following exercises

(3)  Varying exercises of walking with music

(4)  Auditive discrimination with gestures and objects (while seated)

(5)  Varied exercises including the following: Exercises with balls, free movement improvisation, dances or small choreographies to music, call and response games, and singing, accompanied by rhythmic tapping.
